# Joint effects of dietary diversity and intake of edible mushrooms and algae on cognitive impairment among Chinese older adults: a population-based cross-sectional study

**DOI:** 10.3389/fnut.2026.1960150

**Published:** 2026-10-22

**Authors:** Qian Yu, Yingtian Wang, Manshu Yu, Fangyi Chen, Haining Qi, Meng Jiang, Xiaoxiao Wang, Wei Wang

**Affiliations:** 1 Department of Science and Technology, Affiliated Hospital of Nanjing University of Chinese Medicine (Jiangsu Province Hospital of Chinese Medicine), Nanjing, China; 2 School of Nursing, Nanjing University of Chinese Medicine, Nanjing, China; 3 Office of Clinical Research Institute, Affiliated Hospital of Nanjing University of Chinese Medicine (Jiangsu Province Hospital of Chinese Medicine), Nanjing, China; 4 School of Public Health, Nanjing University of Chinese Medicine, Nanjing, China; 5 GCP Center, Affiliated Hospital of Nanjing University of Chinese Medicine (Jiangsu Province Hospital of Chinese Medicine), Nanjing, China

**Keywords:** additive interactions, algae, cognitive impairment, dietary diversity, edible mushrooms, joint effect

## Abstract

**Objective:**

This study aimed to investigate the joint association of dietary diversity (DD) and the intake of edible mushrooms and algae (EMA) with cognitive impairment among Chinese older adults and examine their potential interaction.

**Methods:**

Chinese older adults were recruited from the Chinese Longitudinal Healthy Longevity Survey. Descriptive statistics and multivariable logistic regression models adjusting for demographic, lifestyle, and health-related covariates were employed to evaluate associations. Additive interactions were assessed using the relative excess risk due to interaction (RERI), attributable proportion (AP), and synergy index (SI).

**Results:**

Multivariable logistic regression analysis revealed the joint association of DD and EMA with cognitive impairment among Chinese older adults. In the unadjusted model, compared with participants with poor DD and poor EMA intake (Group 1), both good DD alone (Group 3: OR = 0.653, 95% CI: 0.591–0.873) and good DD combined with good EMA intake (Group 4: OR = 0.466, 95% CI: 0.405–0.779) were associated with lower odds of cognitive impairment (*p* < 0.001). After adjusting for demographic, lifestyle and health-related factors, these inverse associations remained robust (Group 3: OR = 0.766, 95% CI: 0.672–0.897; Group 4: OR = 0.635, 95% CI: 0.525–0.744). Good EMA intake without adequate DD showed no significant association in any adjusted model. No significant multiplicative (*p* = 0.405) or additive (RERI = −0.174, 95% CI: −0.750 to 0.402) interaction was observed between DD and EMA intake.

**Conclusion:**

DD was independently associated with lower odds of cognitive impairment, whereas EMA intake was associated with additional lower odds only within the context of a diverse diet. Public health strategies may promote overall dietary quality alongside targeted mushroom and algae consumption, particularly in high-risk elderly populations.

## 1 Introduction

Cognitive impairment represents a severe global public health challenge, affecting tens of millions of people and imposing substantial socioeconomic burdens (1). Mild cognitive impairment (MCI) is widely recognized as a transitional stage between the cognitive changes of normal aging and the more significant impairment characteristic of dementia (2). According to the World Health Organization (WHO), over 50 million people currently live with dementia worldwide, and a number projected to nearly triple by 2050 (3). The burden of cognitive impairment is particularly acute in China. A study published in The Lancet Public Health revealed that in 2020, the prevalence of cognitive decline and dementia among Chinese adults aged 60 and older was 15.5 and 6.0%, respectively (4). Compounding this issue, Alzheimer’s disease (AD) has now become the fifth leading cause of death for both urban and rural residents, marking it as a rapidly escalating medical and societal crisis.

Cognitive health is influenced by a multitude of factors, including lower educational attainment, hypertension, hearing impairment, smoking, obesity, depression, physical inactivity, diabetes, limited social contact, excessive alcohol consumption, traumatic brain injury, and air pollution (5). While many of these risk factors are non-modifiable, diet stands out as a key evidence-supported modifiable component of lifestyle. Substantial evidence indicates that dietary interventions can not only help stabilize or improve cognitive decline but also protect against dementia and MCI (6). Within the realm of nutrition, the concept of dietary diversity (DD) has gained prominence over a narrow focus on individual foods. DD is defined as the variety of distinct foods or food groups consumed over a specific period (7). Empirical studies have consistently demonstrated that low DD is significantly associated with poorer cognitive performance. Consequently, enhancing DD is recommended as a practical public health strategy to attenuate cognitive decline and lower the incidence of cognitive impairment in older adults, particularly among the oldest old (8–10).

Furthermore, research highlights the significant role of edible mushrooms and algae (EMA) in mitigating the risk of cognitive impairment in older adults (11). Compelling evidence from cohort studies in Japan (12) and Europe (13) has established a consistent association between regular mushroom consumption and a reduced risk of age-related cognitive decline, with analyses revealing a clear dose–response relationship (14). Complementing these findings, studies on fucoxanthin, which is a marine natural product derived from edible algae, have reported its neuroprotective effects against neurodegenerative diseases (15). These benefits are mediated by its ability to cross the blood–brain barrier and execute multimodal mechanisms, including inhibiting Aβ aggregation, suppressing oxidative stress, and reducing neuroinflammation (16).

Despite established individual associations, a critical gap remains in understanding the potential interaction between DD and the intake of EMA on cognitive health. This limitation is significant, as dietary components typically exert synergistic, rather than isolated, biological effects. Additive interaction analysis is a powerful tool in biomedical research for elucidating the complex etiology of multifactorial diseases (17). Therefore, our study aims to investigate the associations of DD and EMA with cognitive impairment among Chinese adults aged 65 years and above, and to formally test for the joint effect and additive interaction. This approach will provide a more integrated and comprehensive scientific basis for understanding the role of combined dietary factors in cognitive aging.

## 2 Materials and methods

### 2.1 Study design and population

This cross-sectional study utilized data from the 2017–2018 wave of the Chinese Longitudinal Healthy Longevity Survey (CLHLS), a nationwide survey conducted in randomly selected counties and cities across 23 provinces and the survey subjects were the elderly aged 65 and above and their adult children aged 35 to 64. Detailed information on the CLHLS is available at: https://opendata.pku.edu.cn. The CLHLS study was approved by the Institutional Review Boards of Peking University and Duke University (IRB00001052-13074). Informed consent was obtained from all participants or their legal representatives (18).

For the present analysis, we initially included all participants (*n* = 15,874) surveyed in the 2017–2018 CLHLS wave. We excluded (1) 103 individuals aged <65 years; (2) 577 with missing data on the intake frequency of any food group (including fresh vegetables, fresh fruits, meat, fish, eggs, legumes and their products, dairy, nuts, tea, EMA); and (3) 3,558 who did not report dementia but had missing items on the Chinese version of the Mini-Mental State Examination (MMSE) or missing years of schooling. The final analytic dataset included 11,636 eligible participants. The detailed screening process is shown in Supplementary Figure 1.

### 2.2 Exposure measures

#### 2.2.1 Dietary diversity scores

Dietary intake was assessed for the following nine food groups: fresh vegetables, fresh fruits, meat, fish, eggs, beans and their products, milk and dairy products, nuts, and tea. The intake frequency of fresh vegetables and fruits was originally recorded using a 4-level scale. For analysis, these responses were dichotomized: a score of 1 was assigned for consumption “every day or almost every day” or “frequently,” and a score of 0 for “occasionally” or “rarely or never”. For the remaining seven food groups, a 5-level scale was used. Similarly, frequencies were dichotomized: a score of 1 was assigned for “almost every day” or “at least once a week but not daily” consumption, and a score of 0 for “at least once a month but not weekly”, “occasionally but not monthly”, or “rarely or never”. The overall DD score was calculated as the sum of the scores from all nine food groups, ranging from 0 to 9. The dichotomization of intake frequency was performed to permit additive interaction analysis, which requires binary exposure categories, and to maintain consistency with previously validated DD constructions in the CLHLS cohort. The median DD score was 5.0 (IQR 3.0–6.0). Restricted cubic spline (RCS) analysis suggested a linear inverse relationship between DD score and cognitive impairment (P for nonlinearity = 0.581; Supplementary Figure 2). Based on the median DD score, participants were categorized into “Good DD” (score ≥ 5) and “Poor DD” (score <5).

#### 2.2.2 Edible mushrooms and algae intake frequency

The intake frequency of EMA was recorded using a 5-level scale, consistent with that of meat (i.e., “almost every day”, “at least once a week but not daily”, “at least once a month but not weekly”, “occasionally but not monthly”, and “rarely or never”). These frequencies were reclassified into 2 levels: a value of 1 was assigned to “almost every day” or “at least once a week but not daily” (representing good EMA intake frequency), and a value of 0 to the remaining categories (representing poor EMA intake frequency), consistent with the reclassification criteria for other food groups.

#### 2.2.3 The joint exposure variable

A joint exposure variable was generated by cross-classifying DD score categories and EMA intake frequency categories, which included four groups: Group 1 (Poor DD score and Poor EMA intake), Group 2 (Poor DD score and Good EMA intake), Group 3 (Good DD score and Poor EMA intake), and Group 4 (Good DD score and Good EMA intake).

### 2.3 Outcome measures

Participants who reported a diagnosis of dementia in the chronic disease section, were directly classified as having cognitive impairment. For those who did not report dementia or whose dementia status was missing, cognitive impairment was determined using the standardized Chinese version of the MMSE, which measures performance across five cognitive domains (9). To account for the confounding effect of education on MMSE performance, we used education-stratified thresholds that have been widely adopted in Chinese epidemiological research and validated in Chinese populations: MMSE ≤17 for illiterate participants (0 years of schooling), ≤20 for those with 1–6 years of schooling (primary education), and ≤24 for those with more than 6 years of schooling (secondary or higher) (19, 20).

### 2.4 Covariates

Potential confounding covariates were extracted from the 2017–2018 CLHLS wave. These included socio-demographic characteristics (age, sex, residence, marital status, education, and occupation), lifestyle and behavioral factors (BMI, smoke status, alcohol consumption, sleep duration, physical exercise, and leisure activity scores), the basic activities of daily living (BADL) score, depressive symptoms defined by the Center for Epidemiological Studies Depression-10 (CESD-10) score with the threshold of 10, and multimorbidity (defined as ≥2 chronic conditions). Covariates were selected based on prior literature and on their statistical association with the exposure groups (*p* < 0.05 in group comparisons), following standard practice in observational studies. Details of the covariate transformations are provided in Supplementary Table 1.

### 2.5 Statistical analysis

Because the proportion of missing values for all covariates (Supplementary Table 2) was below the prespecified 20%, no covariate was excluded due to excessive missingness. Covariates with missing values were imputed using random hot-deck imputation (seed = 316), which preserves the observed distribution of each variable and is appropriate for item-level missingness in large survey datasets. All statistical analyses were performed on the imputed dataset. The descriptive analysis and comparison of participants’ characteristics were implemented based on interaction variables. Continuous variables are presented as mean ± standard deviation (SD) or median interquartile range (IQR), and categorical variables are presented as frequencies and percentage. One-way ANOVA was used for normally distributed continuous variables, the Kruskal-Wallis *H* test for non-normally distributed continuous variables, and the Chi-square test for categorical variables, to compare characteristics across the four exposure groups.

As main analysis, four unconditional logistic regression models were conducted to estimate odds ratios (ORs) and 95% confidence intervals (CIs) for the association between the joint exposure variable and cognitive impairment. Model 1 included the exposure variable only; Model 2 was additionally adjusted for demographic variables (age, sex, residence, marital status and occupation); Model 3 was further adjusted for lifestyle and behavioral factors (BMI, smoking status, alcohol consumption, sleep duration, physical exercise and leisure activity score); Model 4 was further adjusted for health-related variables (BADL score, depressive symptoms defined by CESD-10 score ≥10, and multimorbidity defined as ≥2 chronic conditions). Variables included in above models were those with *p* < 0.05 in the group comparisons.

To test for additive interaction between DD and EMA intake, the relative excess risk due to interaction (RERI), attributable proportion due to interaction (AP), and synergy index (SI) were calculated. The independent associations of DD (Good vs. Poor) and EMA intake frequency (Good vs. Poor) with cognitive impairment were also examined in a fully adjusted model.

Subgroup analyses were performed using the fully adjusted model (Model 4) to estimate the specific association between the joint exposure and cognitive impairment within strata defined by age (<85 vs. ≥85 years), sex (male and female), residence (rural vs. urban), marital status (married vs. others), BMI (<24 vs. ≥24 kg/m^2^), smoking status (never vs. ever/currently), alcohol consumption (never vs. ever/currently), sleep duration (<6 or >8 h, vs. 6–8 h), physical exercise (never vs. ever/currently), leisure activity score (<8 vs. ≥8 points), depressive symptoms (no vs. yes) and multimorbidity (no vs. yes). Because the exposure variable was a four-category joint variable, higher-order interactions between the exposure and subgroup variables were not tested; subgroup analyses were intended to estimate stratum-specific joint association between exposure and cognitive impairment.

Four sensitivity analyses were conducted to verify the robustness of results from main analysis, including Model S1: an alternative categorization of EMA intake frequency (almost every day, at least once per week, and at least once per month grouped into one category; occasionally and never grouped into the other category), the multivariable logistic regression was conducted adjusting for all confounders. Model S2: the multivariable logistic regression was refitted based on dataset imputed by random forest algorithm. Model S3: Considering the high prevalence of cognitive impairment, the multivariable modified Poisson regression was fitted to calculate prevalence ratios (PRs) with 95% CI. Model S4: Cognitive impairment, the dependent variable in multivariable logistic regressions, was redefined as less than 18 of MMSE score.

All statistical analyses and statistical figures were implemented in R software (Version 4.3.1) with the packages haven, VIM, interactionR, rms, randomForest, rqlm. A two-tailed test *p* < 0.05 was considered statistically significant.

## 3 Results

### 3.1 Characteristics of participants

The characteristics of eligible participants were shown in Table 1. A total of 11,636 eligible participants were included in the analysis dataset, mean age was 85.04 ± 11.76 years; 43.32% were male, 48.93% had no formal education, 41.77% were rural residents, 31.53% engaged in regular exercise, and 39.93% had multimorbidity. Notably, only 17.64% maintained optimal dietary patterns (Good DD score + Good EMA intake). Comparative analysis demonstrated that 2,491 participants (21.41%) with cognitive impairment were more likely to be older, female, less educated, and more frequently farmers. They also showed poorer health behaviors, including lower physical activity, longer sleep duration, and suboptimal dietary patterns. These differences were all statistically significant (*p* < 0.05), highlighting the complex interplay between sociodemographic factors, lifestyle choices, and cognitive health in this aging population. The descriptive analysis of DD scores was listed in Supplementary Table 3.

**Table 1:** The characteristics of eligible participants.

Characteristics	Total (*n* = 11,636)	Group 1 (Poor DD + Poor EMA) (*n* = 5,070)	Group 2 (Poor DD + Good EMA) (*n* = 330)	Group 3 (Good DD + Poor EMA) (*n* = 4,184)	Group 4 (Good DD + Good EMA) (*n* = 2052)	*p*-value
Age, years	85.04 ± 11.76	86.26 ± 11.77	83.96 ± 11.82	84.59 ± 11.70	83.10 ± 11.51	<0.001^*^
Sex, *n* (%)						<0.001^#^
Male	5,041 (43.32)	1931 (38.09)	123 (37.27)	1969 (47.06)	1,018 (49.61)	
Female	6,595 (56.68)	3,139 (61.91)	207 (62.73)	2,215 (52.94)	1,034 (50.39)	
Residence, *n* (%)						<0.001^#^
Urban	6,776 (58.23)	2,492 (49.15)	206 (62.42)	2,474 (59.13)	1,604 (78.17)	
Rural	4,860 (41.77)	2,578 (50.85)	124 (37.58)	1710 (40.87)	448 (21.83)	
Marital status, *n* (%)						<0.001^#^
Married	4,889 (42.02)	1843 (36.35)	132 (40.00)	1875 (44.81)	1,039 (50.63)	
Others	6,747 (57.98)	3,227 (63.65)	198 (60.00)	2,309 (55.19)	1,013 (49.37)	
Education, *n* (%)						<0.001^#^
0 year	5,671 (48.93)	3,099 (61.49)	162 (49.09)	1843 (44.20)	567 (27.66)	
1–6 years	3,704 (31.96)	1,497 (29.70)	113 (34.24)	1,450 (34.77)	644 (31.41)	
7–9 years	1,126 (9.72)	272 (5.40)	41 (12.42)	456 (10.94)	357 (17.41)	
≥10 years	1,089 (9.40)	172 (3.41)	14 (4.24)	421 (10.10)	482 (23.51)	
Occupation^⁑^, *n* (%)						<0.001^#^
Category 1	7,118 (61.17)	3,919 (77.30)	182 (55.15)	2,398 (57.31)	619 (30.17)	
Category 2	1759 (15.12)	348 (6.86)	60 (18.18)	754 (18.02)	597 (29.09)	
Category 3	1,385 (11.90)	239 (4.71)	34 (10.30)	523 (12.50)	589 (28.70)	
Category 4	1,374 (11.81)	564 (11.12)	54 (16.36)	509 (12.17)	247 (12.04)	
BMI, kg/m^2^	22.43 ± 4.63	21.92 ± 4.62	22.38 ± 4.71	22.69 ± 4.64	23.19 ± 4.47	<0.001^*^
Smoking status, *n* (%)						<0.001^#^
Never	8,188 (70.37)	3,716 (73.29)	236 (71.52)	2,849 (68.09)	1,387 (67.59)	
Ever	1741 (14.96)	636 (12.54)	43 (13.03)	672 (16.06)	390 (19.01)	
Currently	1707 (14.67)	718 (14.16)	51 (15.45)	663 (15.85)	275 (13.40)	
Alcohol consumption, *n* (%)						<0.001^#^
Never	8,649 (74.33)	3,877 (76.47)	242 (73.33)	3,055 (73.02)	1,475 (71.88)	
Ever	1,342 (11.53)	610 (12.03)	41 (12.42)	450 (10.76)	241 (11.74)	
Currently	1,645 (14.14)	583 (11.50)	47 (14.24)	679 (16.23)	336 (16.37)	
Sleep duration, *n* (%)						<0.001^#^
<6 h	4,221 (36.28)	1969 (38.84)	115 (34.85)	1,465 (35.01)	672 (32.75)	
6–8 h	4,242 (36.46)	1,656 (32.66)	128 (38.79)	1,588 (37.95)	870 (42.40)	
>8 h	3,173 (27.27)	1,445 (28.50)	87 (26.36)	1,131 (27.03)	510 (24.85)	
Physical exercise, *n* (%)						<0.001^#^
Never	7,036 (60.47)	3,628 (71.56)	176 (53.33)	2,457 (58.72)	775 (37.77)	
Ever	931 (8.00)	300 (5.92)	26 (7.88)	339 (8.10)	266 (12.96)	
Currently	3,669 (31.53)	1,142 (22.52)	128 (38.79)	1,388 (33.17)	1,011 (49.27)	
Leisure activity score	10.0 (4.0, 16.0)	8.0 (3.0, 13.0)	8.5 (4.0, 15.8)	11.0 (4.0, 16.0)	13.0 (7.0, 19.0)	<0.001^†^
BADL score	16.81 ± 2.61	16.70 ± 2.76	16.90 ± 2.41	16.87 ± 2.52	16.96 ± 2.42	<0.001^*^
Depressive symptoms, *n* (%)						<0.001^#^
No	3,736 (32.11)	1,506 (29.70)	116 (35.15)	1,371 (32.77)	743 (36.21)	
Yes	7,900 (67.89)	3,564 (70.30)	214 (64.85)	2,813 (67.23)	1,309 (63.79)	
Multimorbidity, *n* (%)						<0.001^#^
No	6,990 (60.07)	3,305 (65.19)	174 (52.73)	2,537 (60.64)	974 (47.47)	
Yes	4,646 (39.93)	1765 (34.81)	156 (47.27)	1,647 (39.36)	1,078 (52.53)	
Cognitive impairment, *n* (%)						<0.001^#^
No	9,145 (78.59)	3,734 (73.65)	260 (78.79)	3,392 (81.07)	1759 (85.72)	
Yes	2,491 (21.41)	1,336 (26.35)	70 (21.21)	792 (18.93)	293 (14.28)	

^*^One-way ANOVA; ^#^Chi-square test; ^†^Kruskal-Wallis *H* test.

^⁑^Occupation: Category 1: Farmer, Category 2: General staff/service staff/workers, Category 3: Professional technicians/doctors/teachers/administrators/soldiers, Category 4: Others.

### 3.2 The association between exposures and outcomes

As shown in Figure 1, in the fully adjusted model (Model 4), compared with participants with poor DD and poor EMA intake (Group 1), those with both good DD and good EMA intake (Group 4) had lower odds of cognitive impairment (OR = 0.635, 95% CI: 0.525–0.744). Good DD alone (Group 3) was also associated with lower odds (OR = 0.766, 95% CI: 0.672–0.897), while good EMA intake without adequate DD (Group 2) showed no significant association (OR = 0.979, 95% CI: 0.693–1.453). The independent associations of DD and EMA intake frequency with cognitive impairment were listed in Supplementary Table 4. Higher DD score was associated with lower odds of cognitive impairment (OR = 0.753, 95% CI: 0.665–0.852, *p* < 0.001), whereas the association for higher EMA intake frequency was marginal and not statistically significant (OR = 0.861, 95% CI: 0.727–1.019, *p* = 0.082).

**Figure 1:**
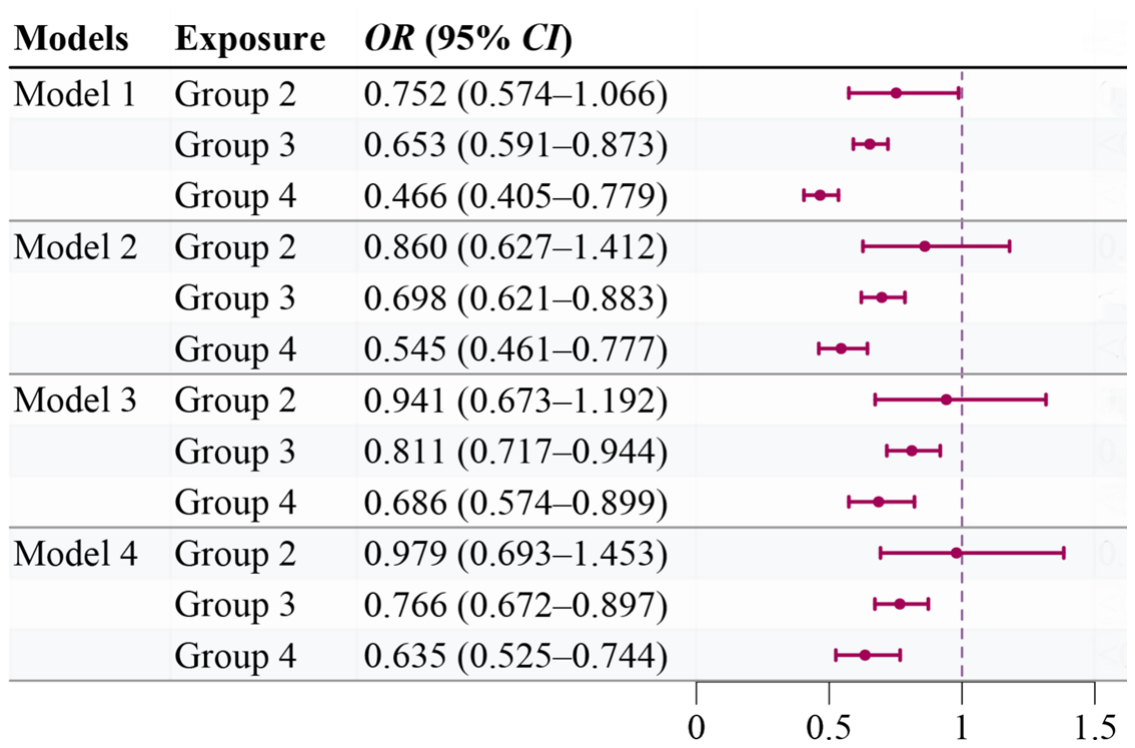
Association between the joint DD–EMA exposure groups and cognitive impairment across four logistic regression models.

As shown in Table 2, interaction analysis revealed no significant multiplicative (*p* = 0.405) or additive (RERI *p* = 0.723) interaction between DD and EMA intake. The RERI was −0.174 (95% CI: −0.750 to 0.402), the AP was −0.110 (95% CI: −0.472 to 0.252), and the SI was 0.768 (95% CI: 0.361–1.637). These findings suggest that the combined association of DD and EMA with cognitive impairment reflects independent contributions rather than an additive interaction.

**Table 2:** Additive and multiplicative interaction between DD and EMA intake on cognitive impairment risk.

Measures	Estimates	LCI	UCI	P
Multiplicative scale	0.846	0.572	1.253	0.405
RERI	−0.174	−0.750	0.402	0.723
AP	−0.110	−0.472	0.252	0.276
SI	0.768	0.361	1.637	0.753

LCI: lower limit of the 95% confidence interval; UCI: upper limit of the 95% confidence interval.

### 3.3 Subgroup analysis

As shown in Figure 2 and Supplementary Table 5, subgroup analyses were performed to estimate the specific association between the joint exposure and cognitive impairment within each stratum. The number and proportion of participants with cognitive impairment within each subgroup and exposure group are presented in Supplementary Table 5. The inverse association for Group 4 was statistically significant in most subgroups, including females, adults aged ≥85 years, rural residents, individuals with BMI < 24 kg/m^2^, never-smokers, and those without depressive symptoms. Conversely, among participants with a history of alcohol consumption, Group 2 was associated with higher odds of cognitive impairment (OR = 2.385, 95% CI: 1.217–4.673). Additive and multiplicative interactions between DD and EMA were further examined within each subgroup, and the results are presented in Supplementary Table 6. Most subgroup analyses showed no significant multiplicative or additive interactions. A few isolated subgroup findings reached nominal significance, including a multiplicative interaction (*p* = 0.005) and an attributable proportion (*p* = 0.029) among participants with a history of alcohol consumption, and a RERI among those without depressive symptoms (*p* = 0.041).

**Figure 2:**
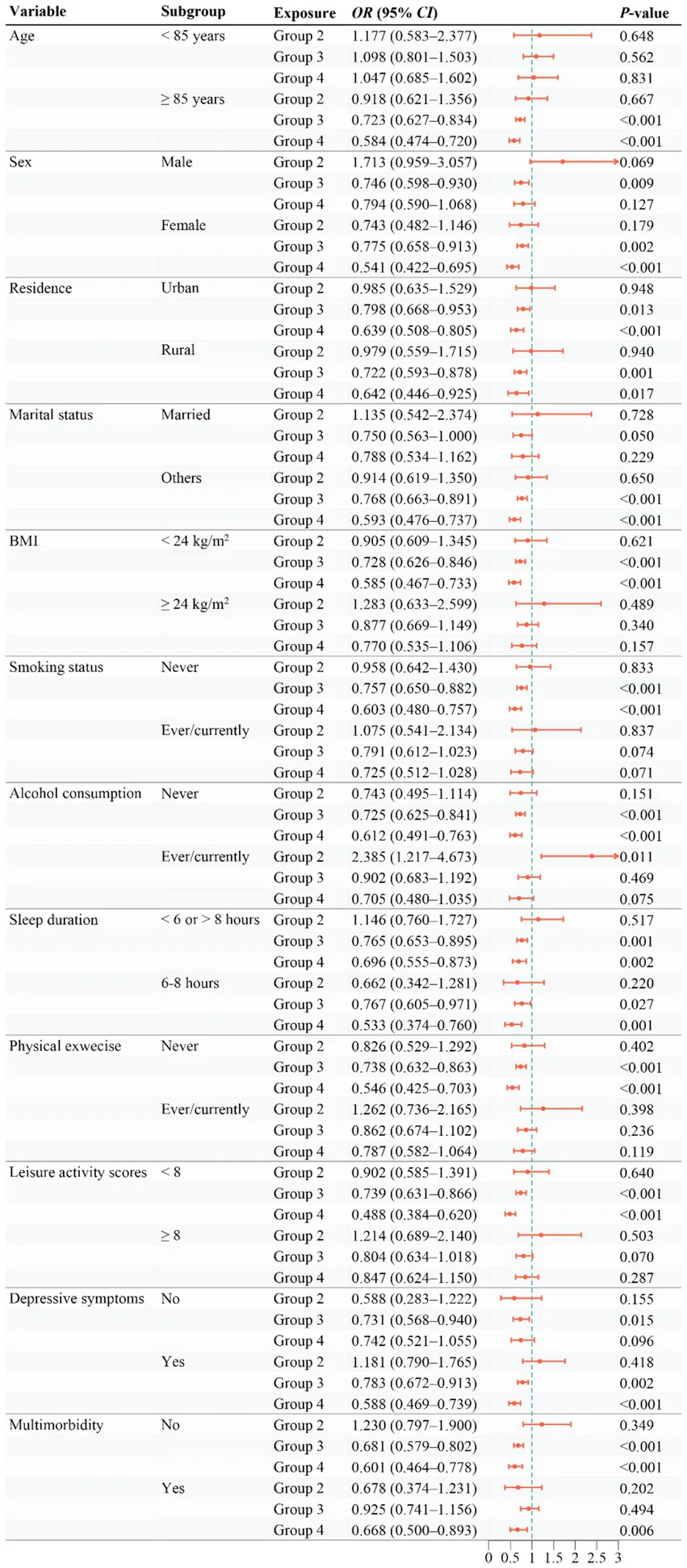
Subgroup-specific associations between the joint DD–EMA exposure groups and cognitive impairment.

### 3.4 Sensitivity analysis

Results of the sensitivity analyses are presented in Figure 3. Model S1 (alternative EMA categorization) yielded results consistent with the primary analysis (Group 3: OR = 0.754, 95% CI: 0.651–0.873; Group 4: OR = 0.664, 95% CI: 0.566–0.779). Model S2 (random forest imputation) produced similar estimates (Group 3: OR = 0.774, 95% CI: 0.679–0.883; Group 4: OR = 0.642, 95% CI: 0.530–0.777). Model S3 (modified Poisson regression) also showed consistent findings (Group 3: PR = 0.886, 95% CI: 0.832–0.944; Group 4: PR = 0.811, 95% CI: 0.732–0.899). Model S4 (MMSE < 18) yielded comparable estimates (Group 3: OR = 0.780, 95% CI: 0.679–0.897; Group 4: OR = 0.604, 95% CI: 0.491–0.744). Additive and multiplicative interactions between DD and EMA were also examined in the sensitivity analyses, and no significant interactions were observed in any model (all *p* > 0.05; Supplementary Table 7).

**Figure 3:**
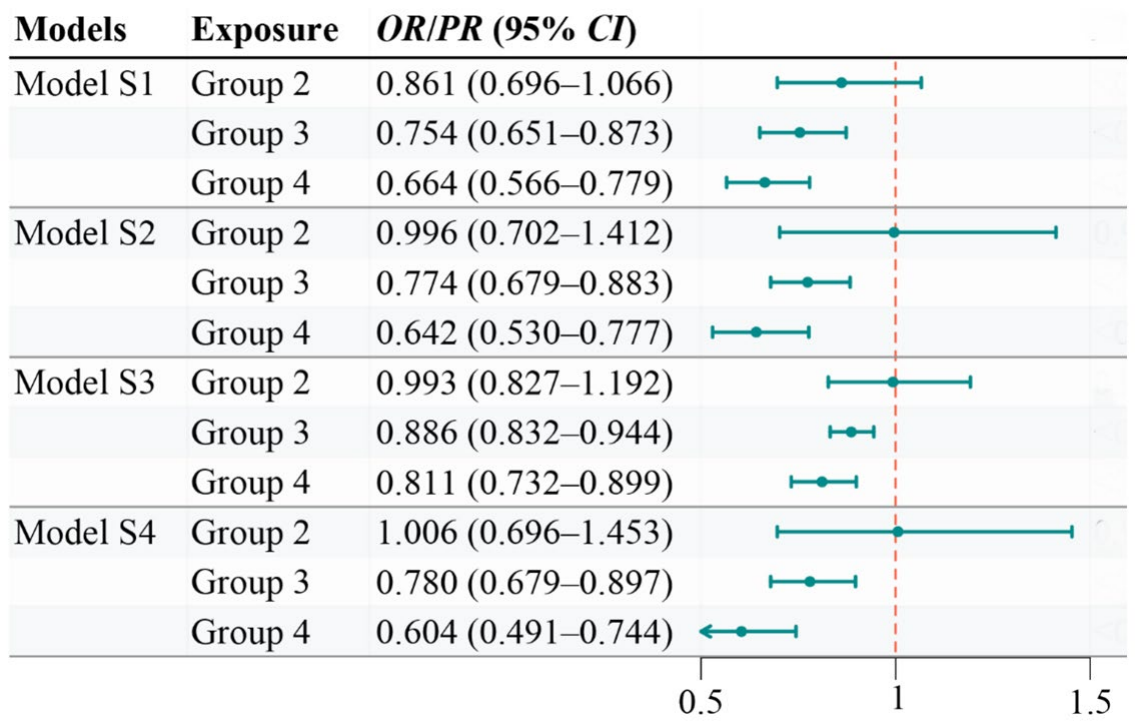
Sensitivity analyses of the association between the joint DD–EMA exposure groups and cognitive impairment.

## 4 Discussion

This population-based cross-sectional study provides evidence regarding the association between dietary patterns and cognitive health among Chinese older adults. Our findings indicate that adequate DD was independently associated with lower odds of cognitive impairment, while regular EMA consumption was associated with additional lower odds only within the context of a diverse diet. Although a combined association was observed, the additive interaction did not reach statistical significance, suggesting that DD and EMA may influence cognitive health through distinct yet complementary biological pathways. The inverse associations appeared consistent across several subgroups, including females, adults aged ≥85 years, and rural residents; however, as noted above, these subgroup findings are descriptive and should not be interpreted as evidence of effect modification.

Prior studies have established significant individual associations between DD, EMA consumption, and cognitive function, findings that align with and contextualize our results. DD serves as a qualitative measure of nutritional adequacy and reflects overall diet quality. Higher DD, characterized by consumption of a wider variety of foods and food groups, is linked to better nutritional status (21, 22), which is a recognized correlate of improved cognitive performance (23). Conversely, insufficient DD may lead to malnutrition, a condition associated with elevated oxidative stress, diminished antioxidant reserves, reduced stress resilience, and impaired homeostasis. These physiological alterations collectively contribute to the decline in the ability to maintain normal brain function in older adults (24). Furthermore, older adults not only experience age-related deterioration in physiological functions tied to nutrient intake but also frequently face oral health issues such as natural tooth loss and denture wear (25). Collectively, these factors render older adults more susceptible to the adverse consequences of inadequate DD compared to their younger counterparts (26). Regarding EMA, evidence indicates that regular mushroom intake helps mitigate the risk of cognitive impairment by reducing the incidence of related conditions such as atherosclerosis, hypertension (27), and diabetes (28), and is associated with better performance across multiple cognitive domains (29–31). Supporting evidence from animal studies further substantiates this association. Zhihui Ma and his colleagues demonstrated that fucoidan, a sulfated polysaccharide isolated from kelp, effectively ameliorates recognition and spatial memory deficits induced by chronic kidney disease in mice (32). In a separate study, administration of an aqueous extract of *P. atrovolvatus* was shown to exert neuroprotective effects against Alzheimer’s disease-related pathology in mouse models, without compromising gut health or memory function (33).

The pivotal finding of our study lies in elucidating the combined association of DD and EMA intake with lower odds of cognitive impairment. This association may be rooted in nutrient synergy, whereby the interactive effects of multiple dietary components may be associated with cognitive health. Accumulating evidence suggests that the cumulative impact of diverse nutrients within holistic dietary patterns surpasses that of any single nutrient (34). The biological activity of many essential nutrients often depends on the presence of complementary nutrients from various food sources. In this context, adequate DD serves as a fundamental prerequisite for achieving nutritional balance and metabolic cooperation, thereby establishing a solid basis for disease prevention (35). This principle is particularly important for understanding the potential cognitive associations of EMA. As natural sources abundant in diverse bioactive compounds with anticancer, antimicrobial, antiviral, antioxidant, antihyperglycemic, and immunomodulatory activities (36–38), their full functional potential depends critically on the overall dietary context. For example, the bioavailability of lipid-soluble bioactive compounds in EMA requires the presence of dietary lipids for efficient absorption (39). Meanwhile, their unique dietary fibers, such as alginate, depend on a diverse gut microbiota for fermentation, a microbial ecosystem which in turn relies on a regular supply of varied dietary fibers from different plant-based foods to maintain its diversity (40, 41). Thus, the potential cognitive associations derived from these bioactivities are contingent upon a supportive dietary environment characterized by overall diversity. While cognitive health is influenced by macronutrients, it is also susceptible to impairment from deficiencies in specific micronutrients. Micronutrients such as B vitamins, iron, and omega-3 fatty acids are critical for cognitive function (42). For example, B vitamins, which are rich in algae, are essential for DNA methylation and help prevent the accumulation of homocysteine, which is linked to cognitive decline (43). Among them, vitamin B_12_ plays a key role in neural activation and antioxidant defense (44). Therefore, the potential cognitive associations of EMA are best realized when embedded within a “nutrient network” constituted by a diverse diet.

This study has several limitations. First, the cross-sectional design precludes causal inference, and reverse causation cannot be excluded. Cognitive impairment may affect participants’ ability to accurately recall dietary intake, potentially leading to systematic under- or over-reporting of food variety among individuals with cognitive impairment. Depending on the direction of misreporting, this could either attenuate or inflate the observed association. Second, our DD score was derived from the intake frequency of nine food groups rather than from the variety of individual items within each group; this measure therefore reflects food group variety and may not fully capture within-group dietary diversity. Third, the dichotomization of intake frequency into binary categories may reduce information and statistical power; this approach was chosen to permit additive interaction analysis and to align with previously validated DD constructions in the CLHLS cohort. Fourth, we were unable to adjust for total energy intake, as this information is not available in the CLHLS dataset. Fifth, the simplified food frequency questionnaire (FFQ) employed in the CLHLS survey captured food group consumption frequency but not precise intake amounts, limiting our ability to examine dose–response relationships. Sixth, among participants with a documented dementia diagnosis but missing MMSE data, the severity of dementia could not be determined. Finally, although extensive covariate adjustment was performed, residual confounding due to unmeasured factors and self-reported data cannot be ruled out. Prospective cohort studies are needed to validate these preliminary cross-sectional findings.

## 5 Conclusion

In this large-scale population-based study, DD was independently associated with lower odds of cognitive impairment in Chinese older adults. Mushrooms and algae appeared to be associated with additional lower odds only within the context of a diverse diet. These findings support food-based dietary guidelines that emphasize overall dietary quality, while further prospective studies are needed to confirm these cross-sectional associations.

## Data Availability

The datasets presented in this study can be found in online repositories. The names of the repository/repositories and accession number(s) can be found at: Detailed information on the CLHLS is available at: https://opendata.pku.edu.cn.
