# Supplementary material for: Joint effects of dietary diversity and intake of edible mushrooms and algae on cognitive impairment among Chinese older adults: a population-based cross-sectional study
Source: Front Nutr. 2026 Oct 22;13:1960150. doi: 10.3389/fnut.2026.1960150 (PMC13761222; doi:10.3389/fnut.2026.1960150)
Supplement: Supplementary file 2 [file Supplementary_file_2.docx]

**Supplementary Figure 2**

**Restricted cubic spline analysis of the association between DD score and cognitive impairment**

Supplementary Figure 2 Restricted cubic spline analysis of the association between DD score and cognitive impairment
