# Supplementary material for: Joint effects of dietary diversity and intake of edible mushrooms and algae on cognitive impairment among Chinese older adults: a population-based cross-sectional study
Source: Front Nutr. 2026 Oct 22;13:1960150. doi: 10.3389/fnut.2026.1960150 (PMC13761222; doi:10.3389/fnut.2026.1960150)
Supplement: Supplementary file 3 [file Table_1.docx]

**Supplementary Table 1**

**The names, labels and transformation of variables in the study**

Supplementary Table 1 The names, labels and transformation of variables in the study

| Names of variables | Labels | Source or definitions |
| --- | --- | --- |
| Age | Current Age | Validated age at the time of survey |
| Sex | 1=male, 2=female | Sex of participants |
| Residence | 1=urban (city or town), 2=rural | the current residential area of the interviewed elderly (city, town, rural) |
| Marital status | 1=married, 2= Others (divorced, widowed, never married) | current marital status |
| Education | 0=0 years, 1=1-6 years,  3=7-9 years, 4= 10 years and over | years of schooling for participants |
|  |  |  |
| Occupation | 1=Farmer, 2=General staff/service staff/workers  3=Professional technicians/  doctors/teachers/  administrators/soldiers, 4=Others | main occupation before age 60 |
| BMI | Continues variable | Weight/(height)^2 |
| Smoking status | 1=never, 2= ever, 3=currently | smoke or not at present?  smoked or not in the past? |
| Alcohol status | 1=never, 2= ever, 3=currently | drink or not at present?  drank or not in the past? |
| Sleep duration | 1= less than 6 hours, 2= from 6 to 8 hours, 3= more than 8 hours | how many hours do you usually sleep each day? |
| Physical exercise | 1=never, 2= ever, 3=currently | exercise or not at present?  exercised or not in the past? |
| Leisure activity score | Each item was scored as follows: 0 (never), 1 (not monthly, but sometimes), 2 (not weekly, but at least once for a month), 3 (not daily, but once for a week) and 4 (almost everyday). The total score ranges from 0 to 44. | 1. do you do house work at present?  2. do you take outdoor activities? (tai chi chuan, square dance, series, interact with friends and others)  3. do you do garden work?  4. do you read newspapers/books at present?  5. do you raise domestic animals/pets at present?  6. do you play cards/mah-jongg at present?  7. do you watch tv or listen to radio at present?  8. do you take part in some social activities at present? |
| Basic ability of daily living | Each item was scored as follows: 1 (more than one part assistance), 2 (one part assistance), and 3 (without assistance). The total score of the activity score ranges from 6 to 18. | Feeding, Indoor transferring, Dressing, Continence, Bathing, Toileting |
| Depressive symptoms | Depressive symptoms, binary categorical variable, defined as a score ≥10 on the 10-item Center for Epidemiologic Studies Depression Scale. Each item was scored as follows: 0 (rarely or none of the time, <1 day), 1 (some or little of the time, 1–2 days), 2 (occasionally or a moderate amount of the time, 3–4 days), and 3 (most or all of the time, 5–7 days). The total score of the CESD-10 ranges from 0 to 30 | 1. Are you full of hope for future life?  2. Do you feel as happy as you are when you are young?  3. Is it difficult to concentrate when you are doing things now  4. Do you feel unable to continue your life?  5. Are you feeling sad or depressed?  6. Are you nervous and scared?  7. Do you think that the older you are, the less useful you are, and the hard work  8. Do you feel lonely?  9. Are you worried about some small things?  10.How is your sleep quality now? |
| Multimorbidity | 1= yes , 0=no | The number of common chronic condition (≥2 and <2), including hypertension, diabetes, heart disease, stroke, bronchitis/emphysema/pneumonia/asthma, tuberculosis, cataract, glaucoma, cancer, gastric or duodenal ulcer, Parkinson’s disease, arthritis, epilepsy, cholecystitis/cholelithiasis, dyslipidemia, rheumatism or rheumatoid disease, chronic nephritis, and hepatitis |
