# Supplementary material for: Joint effects of dietary diversity and intake of edible mushrooms and algae on cognitive impairment among Chinese older adults: a population-based cross-sectional study
Source: Front Nutr. 2026 Oct 22;13:1960150. doi: 10.3389/fnut.2026.1960150 (PMC13761222; doi:10.3389/fnut.2026.1960150)
Supplement: Supplementary file 4 [file Table_2.docx]

**Supplementary Table 2**

**The covariate and the proportion of missing value**

Supplementary Table 2 The covariate and the proportion of missing value

| Variable | Proportion (%) |
| --- | --- |
| Chronic conditions |  |
| Cancer | 16.03 |
| Dyslipidemia | 12.32 |
| Glaucoma | 12.26 |
| Chronic lung disease | 12.17 |
| Nephritis | 12.12 |
| Hepatitis | 11.94 |
| Gallbladder | 11.68 |
| Gastric ulcer | 11.64 |
| Rheumatism | 11.63 |
| Tuberculosis | 11.45 |
| Parkinson disease | 11.45 |
| Epilepsy | 11.33 |
| Cataract | 11.30 |
| Dementia | 10.86 |
| Arthritis | 10.85 |
| Stroke | 10.50 |
| Diabetes | 10.46 |
| Heart disease | 10.06 |
| Hypertension | 6.92 |
| CESD-10 |  |
| are you full of hope for future life? | 12.13 |
| do you feel as happy as you are when you are young? | 11.97 |
| is it difficult to concentrate when you are doing things now | 10.20 |
| do you feel unable to continue your life? | 10.08 |
| are you feeling sad or depressed? | 9.08 |
| are you nervous and scared? | 9.04 |
| do you think that the older you are, the less useful you are, and the hard work | 8.95 |
| do you feel lonely? | 8.45 |
| are you worried about some small things? | 8.13 |
| how is your sleep quality now? | 5.17 |
| Anthropometric measurements |  |
| Height | 5.05 |
| Weight | 4.55 |
| Lifestyle behaviors |  |
| Sleep duration | 3.50 |
| drank or not in the past? | 1.80 |
| drink or not at present? | 1.38 |
| smoked or not in the past? | 1.34 |
| smoke or not at present? | 0.88 |
| exercised or not in the past? | 1.23 |
| exercise or not at present? | 1.11 |
| Basic activities of daily living |  |
| Feeding | 2.01 |
| Indoor transferring | 0.86 |
| Dressing | 0.74 |
| Continence | 0.40 |
| Bathing | 0.37 |
| toileting | 0.32 |
| Demographic characteristics |  |
| Occupation | 1.40 |
| Marital status | 0.89 |
| Years of schooling | 0.40 |
| MMSE score | 1.20 |
| Leisure activity score |  |
| do you take part in some social activities at present? | 0.95 |
| tai chi chuan | 0.57 |
| other outdoor activities | 0.54 |
| square dance | 0.51 |
| series, interact with friends | 0.50 |
| do you do house work at present? | 0.38 |
| do you raise domestic animals/pets at present? | 0.23 |
| do you watch tv or listen to radio at present ? | 0.22 |
| do you read newspapers/books at present? | 0.20 |
| do you do garden work | 0.16 |
| do you play cards/mah-jongg at present? | 0.16 |
