# Supplementary material for: Joint effects of dietary diversity and intake of edible mushrooms and algae on cognitive impairment among Chinese older adults: a population-based cross-sectional study
Source: Front Nutr. 2026 Oct 22;13:1960150. doi: 10.3389/fnut.2026.1960150 (PMC13761222; doi:10.3389/fnut.2026.1960150)
Supplement: Supplementary file 5 [file Table_3.docx]

**Supplementary Table 3**

**The descriptive analysis of dietary diversity scores**

Supplementary Table 3 The descriptive analysis of dietary diversity scores

| Dietary diversity score | Range (min-max) | Median (IQR) | Mean±SD |
| --- | --- | --- | --- |
| Total | 0-9.0 | 5.0 (3.0-6.0) | 4.7±2.0 |
| Group 1 | 0-4.0 | 3.0 (2.0-4.0) | 2.8±1.1 |
| Group 2 | 0-4.0 | 4.0 (3.0-4.0) | 3.3±0.8 |
| Group 3 | 5.0-9.0 | 6.0 (5.0-7.0) | 6.0±1.0 |
| Group 4 | 5.0-9.0 | 7.0 (6.0-8.0) | 6.9±1.2 |

IQR: interquartile range; SD: standard deviance
