# Supplementary material for: Joint effects of dietary diversity and intake of edible mushrooms and algae on cognitive impairment among Chinese older adults: a population-based cross-sectional study
Source: Front Nutr. 2026 Oct 22;13:1960150. doi: 10.3389/fnut.2026.1960150 (PMC13761222; doi:10.3389/fnut.2026.1960150)
Supplement: Supplementary file 6 [file Table_4.docx]

**Supplementary Table 4**

**Independent associations of DD and EMA intake with cognitive impairment**

Supplementary Table 4 Independent associations of DD and EMA intake with cognitive impairment

| Variable | β | SE | OR (95% CI) | z | P |
| --- | --- | --- | --- | --- | --- |
| DD |  |  |  |  |  |
| Poor | Reference |  |  |  |  |
| Good | -0.284 | 0.063 | 0.753 (0.665-0.852) | -4.476 | < 0.001 |
| EMA intake frequency |  |  |  |  |  |
| Poor | Reference |  |  |  |  |
| Good | -0.150 | 0.086 | 0.861 (0.727-1.019) | -1.741 | 0.082 |
| Age | 0.072 | 0.004 | 1.075 (1.067-1.083) | 18.964 | < 0.001 |
| Sex |  |  |  |  |  |
| Male | Reference |  |  |  |  |
| Female | 0.108 | 0.074 | 1.114 (0.964-1.287) | 1.464 | 0.143 |
| Residence |  |  |  |  |  |
| Urban | Reference |  |  |  |  |
| Rural | 0.067 | 0.063 | 1.070 (0.946-1.21) | 1.069 | 0.285 |
| Marital status |  |  |  |  |  |
| Married | Reference |  |  |  |  |
| Others | 0.122 | 0.083 | 1.129 (0.96-1.328) | 1.467 | 0.142 |
| Occupation⁑ |  |  |  |  |  |
| Category 1 | Reference |  |  |  |  |
| Category 2 | 0.186 | 0.098 | 1.204 (0.994-1.459) | 1.897 | 0.058 |
| Category 3 | 0.090 | 0.116 | 1.094 (0.871-1.375) | 0.774 | 0.439 |
| Category 4 | 0.000 | 0.089 | 1.000 (0.839-1.191) | -0.002 | 0.998 |
| BMI | -0.007 | 0.007 | 0.993 (0.980-1.006) | -1.111 | 0.267 |
| Smoking status |  |  |  |  |  |
| Never | Reference |  |  |  |  |
| Ever | 0.078 | 0.096 | 1.081 (0.896-1.303) | 0.813 | 0.416 |
| Currently | 0.090 | 0.104 | 1.095 (0.893-1.341) | 0.872 | 0.383 |
| Alcohol consumption |  |  |  |  |  |
| Never | Reference |  |  |  |  |
| Ever | 0.070 | 0.099 | 1.072 (0.882-1.303) | 0.699 | 0.485 |
| Currently | -0.079 | 0.102 | 0.924 (0.756-1.128) | -0.777 | 0.437 |
| Sleep duration |  |  |  |  |  |
| < 6 hours | Reference |  |  |  |  |
| 6-8 hours | -0.051 | 0.072 | 0.951 (0.825-1.095) | -0.700 | 0.484 |
| > 8 hours | 0.129 | 0.071 | 1.138 (0.991-1.307) | 1.829 | 0.067 |
| Physical exercise |  |  |  |  |  |
| Never | Reference |  |  |  |  |
| Ever | -0.176 | 0.099 | 0.839 (0.691-1.018) | -1.778 | 0.075 |
| Currently | -0.099 | 0.082 | 0.906(0.772-1.063) | -1.212 | 0.225 |
| Leisure activity score | -0.098 | 0.006 | 0.907(0.895-0.918) | -15.386 | < 0.001 |
| BADL score | -0.248 | 0.012 | 0.78(0.763-0.798) | -21.491 | < 0.001 |
| Depressive symptoms |  |  |  |  |  |
| No | Reference |  |  |  |  |
| Yes | 0.235 | 0.065 | 1.265 (1.114-1.436) | 3.631 | < 0.001 |
| Multimorbidity |  |  |  |  |  |
| No | Reference |  |  |  |  |
| Yes | -0.027 | 0.064 | 0.973 (0.859-1.103) | -0.421 | 0.673 |

⁑Category 1: Farmer, Category 2: General staff/service staff/workers, Category 3: Professional technicians/doctors/teachers/administrators/soldiers, Category 4: Others
