# Supplementary material for: Joint effects of dietary diversity and intake of edible mushrooms and algae on cognitive impairment among Chinese older adults: a population-based cross-sectional study
Source: Front Nutr. 2026 Oct 22;13:1960150. doi: 10.3389/fnut.2026.1960150 (PMC13761222; doi:10.3389/fnut.2026.1960150)
Supplement: Supplementary file 7 [file Table_5.docx]

**Supplementary Table 5**

**Number and proportion of cognitive impairment in subgroups**

Supplementary Table 5 Number and proportion of cognitive impairment in subgroups

| Variable | Subgroup | Exposure | N | Cognitive impairment (%) |
| --- | --- | --- | --- | --- |
| Age | < 85 years | Group 1 | 2281 | 117 (5.13) |
|  |  | Group 2 | 181 | 10 (5.52) |
|  |  | Group 3 | 2136 | 91 (4.26) |
|  |  | Group 4 | 1151 | 42 (3.65) |
|  | ≥ 85 years | Group 1 | 2789 | 1219 (43.71) |
|  |  | Group 2 | 149 | 60 (40.27) |
|  |  | Group 3 | 2048 | 701 (34.23) |
|  |  | Group 4 | 901 | 251 (27.86) |
| Sex | Male | Group 1 | 1931 | 385 (19.94) |
|  |  | Group 2 | 123 | 28 (22.76) |
|  |  | Group 3 | 1969 | 253 (12.85) |
|  |  | Group 4 | 1018 | 118 (11.59) |
|  | Female | Group 1 | 3139 | 951 (30.30) |
|  |  | Group 2 | 207 | 42 (20.29) |
|  |  | Group 3 | 2215 | 539 (24.33) |
|  |  | Group 4 | 1034 | 175 (16.92) |
| Residence | Urban | Group 1 | 2492 | 666 (26.73) |
|  |  | Group 2 | 206 | 45 (21.84) |
|  |  | Group 3 | 2474 | 474 (19.16) |
|  |  | Group 4 | 1604 | 234 (14.59) |
|  | Rural | Group 1 | 2578 | 670 (25.99) |
|  |  | Group 2 | 124 | 25 (20.16) |
|  |  | Group 3 | 1710 | 318 (18.60) |
|  |  | Group 4 | 448 | 59 (13.17) |
| Marital status | Married | Group 1 | 1843 | 192 (10.42) |
|  |  | Group 2 | 132 | 12 (9.09) |
|  |  | Group 3 | 1875 | 112 (5.97) |
|  |  | Group 4 | 1039 | 56 (5.39) |
|  | Others | Group 1 | 3227 | 1144 (35.45) |
|  |  | Group 2 | 198 | 58 (29.29) |
|  |  | Group 3 | 2309 | 680 (29.45) |
|  |  | Group 4 | 1013 | 237 (23.40) |
| BMI | < 24 kg/m^2^ | Group 1 | 3747 | 1097 (29.28) |
|  |  | Group 2 | 228 | 55 (24.12) |
|  |  | Group 3 | 2741 | 593 (21.63) |
|  |  | Group 4 | 1234 | 212 (17.18) |
|  | ≥ 24 kg/m^2^ | Group 1 | 1323 | 239 (18.07) |
|  |  | Group 2 | 102 | 15 (14.71) |
|  |  | Group 3 | 1443 | 199 (13.79) |
|  |  | Group 4 | 818 | 81 (9.90) |
| Smoking status | Never | Group 1 | 3716 | 1067 (28.71) |
|  |  | Group 2 | 236 | 54 (22.88) |
|  |  | Group 3 | 2849 | 607 (21.31) |
|  |  | Group 4 | 1387 | 209 (15.07) |
|  | Ever/currently | Group 1 | 1354 | 269 (19.87) |
|  |  | Group 2 | 94 | 16 (17.02) |
|  |  | Group 3 | 1335 | 185 (13.86) |
|  |  | Group 4 | 665 | 84 (12.63) |
| Alcohol consumption | Never | Group 1 | 3877 | 1103 (28.45) |
|  |  | Group 2 | 242 | 49 (20.25) |
|  |  | Group 3 | 3055 | 633 (20.72) |
|  |  | Group 4 | 1475 | 224 (15.19) |
|  | Ever/currently | Group 1 | 1193 | 233 (19.53) |
|  |  | Group 2 | 88 | 21 (23.86) |
|  |  | Group 3 | 1129 | 159 (14.08) |
|  |  | Group 4 | 577 | 69 (11.96) |
| Sleep duration | < 6 or > 8 hours | Group 1 | 3414 | 969 (28.38) |
|  |  | Group 2 | 202 | 54 (26.73) |
|  |  | Group 3 | 2596 | 539 (20.76) |
|  |  | Group 4 | 1182 | 212 (17.94) |
|  | 6-8 hours | Group 1 | 1656 | 367 (22.16) |
|  |  | Group 2 | 128 | 16 (12.50) |
|  |  | Group 3 | 1588 | 253 (15.93) |
|  |  | Group 4 | 870 | 81 (9.31) |
| Physical exercise | Never | Group 1 | 3628 | 1100 (30.32) |
|  |  | Group 2 | 176 | 45 (25.57) |
|  |  | Group 3 | 2457 | 573 (23.32) |
|  |  | Group 4 | 775 | 161 (20.77) |
|  | Ever/currently | Group 1 | 1442 | 236 (16.37) |
|  |  | Group 2 | 154 | 25 (16.23) |
|  |  | Group 3 | 1727 | 219 (12.68) |
|  |  | Group 4 | 1277 | 132 (10.34) |
| Leisure activity scores | < 8 points | Group 1 | 2386 | 1139 (47.74) |
|  |  | Group 2 | 120 | 53 (44.17) |
|  |  | Group 3 | 1429 | 627 (43.88) |
|  |  | Group 4 | 540 | 201 (37.22) |
|  | ≥ 8 points | Group 1 | 2684 | 197 (7.34) |
|  |  | Group 2 | 210 | 17 (8.10) |
|  |  | Group 3 | 2755 | 165 (5.99) |
|  |  | Group 4 | 1512 | 92 (6.08) |
| Depressive symptoms | No | Group 1 | 1506 | 348 (23.11) |
|  |  | Group 2 | 116 | 14 (12.07) |
|  |  | Group 3 | 1371 | 209 (15.24) |
|  |  | Group 4 | 743 | 97 (13.06) |
|  | Yes | Group 1 | 3564 | 988 (27.72) |
|  |  | Group 2 | 214 | 56 (26.17) |
|  |  | Group 3 | 2813 | 583 (20.73) |
|  |  | Group 4 | 1309 | 196 (14.97) |
| Multimorbidity | No | Group 1 | 3305 | 941 (28.47) |
|  |  | Group 2 | 174 | 47 (27.01) |
|  |  | Group 3 | 2537 | 479 (18.88) |
|  |  | Group 4 | 974 | 140 (14.37) |
|  | Yes | Group 1 | 1765 | 395 (22.38) |
|  |  | Group 2 | 156 | 23 (14.74) |
|  |  | Group 3 | 1647 | 313 (19.00) |
|  |  | Group 4 | 1078 | 153 (14.19) |
