# Supplementary material for: Joint effects of dietary diversity and intake of edible mushrooms and algae on cognitive impairment among Chinese older adults: a population-based cross-sectional study
Source: Front Nutr. 2026 Oct 22;13:1960150. doi: 10.3389/fnut.2026.1960150 (PMC13761222; doi:10.3389/fnut.2026.1960150)
Supplement: Supplementary file 8 [file Table_6.docx]

**Supplementary Table 6**

**Interaction effect of DD and EMA intake on cognitive impairment risk among subgroups**

Supplementary Table 6 Interaction effect of DD and EMA intake on cognitive impairment risk among subgroups

| Variable | Subgroup | Measures | Estimates | LCI | UCI | P |
| --- | --- | --- | --- | --- | --- | --- |
| Age | < 85 years | Multiplicative scale | 0.810 | 0.362 | 1.812 | 0.609 |
|  |  | RERI | -0.218 | -1.124 | 0.689 | 0.681 |
|  |  | AP | -0.228 | -1.156 | 0.700 | 0.315 |
|  |  | SI | - | - | - | - |
|  | ≥ 85 years | Multiplicative scale | 0.881 | 0.567 | 1.368 | 0.572 |
|  |  | RERI | -0.096 | -0.753 | 0.561 | 0.613 |
|  |  | AP | -0.056 | -0.439 | 0.326 | 0.387 |
|  |  | SI | 0.881 | 0.393 | 1.975 | 0.621 |
| Sex | Male | Multiplicative scale | 0.622 | 0.326 | 1.187 | 0.150 |
|  |  | RERI | -0.836 | -2.145 | 0.472 | 0.894 |
|  |  | AP | -0.664 | -1.678 | 0.350 | 0.100 |
|  |  | SI | 0.237 | 0.057 | 0.991 | 0.975 |
|  | Female | Multiplicative scale | 0.940 | 0.572 | 1.545 | 0.808 |
|  |  | RERI | 0.043 | -0.619 | 0.706 | 0.449 |
|  |  | AP | 0.023 | -0.336 | 0.383 | 0.449 |
|  |  | SI | 1.054 | 0.460 | 2.414 | 0.451 |
| Residence | Urban | Multiplicative scale | 0.813 | 0.498 | 1.327 | 0.407 |
|  |  | RERI | -0.226 | -0.954 | 0.502 | 0.728 |
|  |  | AP | -0.144 | -0.607 | 0.319 | 0.271 |
|  |  | SI | 0.714 | 0.285 | 1.792 | 0.763 |
|  | Rural | Multiplicative scale | 0.910 | 0.463 | 1.788 | 0.783 |
|  |  | RERI | -0.090 | -1.040 | 0.860 | 0.574 |
|  |  | AP | -0.051 | -0.662 | 0.546 | 0.425 |
|  |  | SI | 0.860 | 0.206 | 3.600 | 0.581 |
| Marital status | Married | Multiplicative scale | 0.925 | 0.405 | 2.112 | 0.854 |
|  |  | RERI | -0.123 | -1.250 | 1.003 | 0.585 |
|  |  | AP | -0.097 | -0.979 | 0.785 | 0.415 |
|  |  | SI | 0.685 | 0.040 | 11.665 | 0.603 |
|  | Others | Multiplicative scale | 0.844 | 0.541 | 1.316 | 0.454 |
|  |  | RERI | -0.151 | -0.806 | 0.503 | 0.674 |
|  |  | AP | -0.090 | -0.475 | 0.296 | 0.324 |
|  |  | SI | 0.820 | 0.380 | 1.770 | 0.693 |
| BMI | < 24 kg/m^2^ | Multiplicative scale | 0.888 | 0.563 | 1.399 | 0.607 |
|  |  | RERI | -0.083 | -0.747 | 0.582 | 0.596 |
|  |  | AP | -0.048 | -0.435 | 0.339 | 0.403 |
|  |  | SI | 0.895 | 0.390 | 2.056 | 0.602 |
|  | ≥ 24 kg/m^2^ | Multiplicative scale | 0.684 | 0.314 | 1.492 | 0.340 |
|  |  | RERI | -0.507 | -1.755 | 0.742 | 0.787 |
|  |  | AP | -0.390 | -1.337 | 0.557 | 0.210 |
|  |  | SI | 0.372 | 0.068 | 2.017 | 0.874 |
| Smoking status | Never | Multiplicative scale | 0.831 | 0.526 | 1.314 | 0.429 |
|  |  | RERI | -0.186 | -0.876 | 0.505 | 0.701 |
|  |  | AP | -0.112 | -0.524 | 0.300 | 0.298 |
|  |  | SI | 0.780 | 0.349 | 1.742 | 0.728 |
|  | Ever/currently | Multiplicative scale | 0.853 | 0.397 | 1.832 | 0.684 |
|  |  | RERI | -0.193 | -1.278 | 0.891 | 0.637 |
|  |  | AP | -0.140 | -0.921 | 0.640 | 0.362 |
|  |  | SI | 0.662 | 0.101 | 4.326 | 0.667 |
| Alcohol consumption | Never | Multiplicative scale | 1.137 | 0.719 | 1.798 | 0.583 |
|  |  | RERI | 0.236 | -0.301 | 0.773 | 0.194 |
|  |  | AP | 0.145 | -0.189 | 0.478 | 0.198 |
|  |  | SI | 1.594 | 0.384 | 6.602 | 0.260 |
|  | Ever/currently | Multiplicative scale | 0.328 | 0.151 | 0.709 | 0.005 |
|  |  | RERI | -2.243 | -4.701 | 0.215 | 0.963 |
|  |  | AP | -1.582 | -3.221 | 0.057 | 0.029 |
|  |  | SI | 0.157 | 0.048 | 0.512 | 0.999 |
| Sleep duration | < 6 or > 8 hours | Multiplicative scale | 0.795 | 0.498 | 1.269 | 0.336 |
|  |  | RERI | -0.307 | -1.033 | 0.419 | 0.796 |
|  |  | AP | -0.214 | -0.711 | 0.284 | 0.200 |
|  |  | SI | 0.587 | 0.225 | 1.532 | 0.862 |
|  | 6-8 hours | Multiplicative scale | 1.051 | 0.500 | 2.208 | 0.895 |
|  |  | RERI | 0.197 | -0.715 | 1.109 | 0.336 |
|  |  | AP | 0.105 | -0.385 | 0.595 | 0.338 |
|  |  | SI | 1.290 | 0.306 | 5.109 | 0.359 |
| Physical exercise | Never | Multiplicative scale | 0.895 | 0.536 | 1.497 | 0.674 |
|  |  | RERI | -0.033 | -0.771 | 0.705 | 0.535 |
|  |  | AP | -0.018 | -0.420 | 0.384 | 0.465 |
|  |  | SI | 0.961 | 0.411 | 2.249 | 0.536 |
|  | Ever/currently | Multiplicative scale | 0.723 | 0.394 | 1.327 | 0.295 |
|  |  | RERI | -0.429 | -1.353 | 0.495 | 0.818 |
|  |  | AP | -0.337 | -1.057 | 0.382 | 0.179 |
|  |  | SI | 0.388 | 0.089 | 1.694 | 0.896 |
| Leisure activity scores | < 8 points | Multiplicative scale | 0.732 | 0.446 | 1.201 | 0.217 |
|  |  | RERI | -0.314 | -1.183 | 0.555 | 0.760 |
|  |  | AP | -0.153 | -0.571 | 0.266 | 0.237 |
|  |  | SI | 0.770 | 0.412 | 1.440 | 0.793 |
|  | ≥ 8 points | Multiplicative scale | 0.868 | 0.462 | 1.631 | 0.661 |
|  |  | RERI | -0.201 | -1.058 | 0.656 | 0.677 |
|  |  | AP | -0.170 | -0.891 | 0.551 | 0.322 |
|  |  | SI | 0.473 | 0.045 | 4.962 | 0.734 |
| Depressive symptoms | No | Multiplicative scale | 1.727 | 0.772 | 3.862 | 0.184 |
|  |  | RERI | 0.571 | -0.073 | 1.215 | 0.041 |
|  |  | AP | 0.423 | -0.074 | 0.921 | 0.048 |
|  |  | SI | - | - | - | - |
|  | Yes | Multiplicative scale | 0.636 | 0.402 | 1.008 | 0.054 |
|  |  | RERI | -0.638 | -1.510 | 0.234 | 0.924 |
|  |  | AP | -0.376 | -0.875 | 0.124 | 0.070 |
|  |  | SI | 0.523 | 0.275 | 0.994 | 0.976 |
| Multimorbidity | No | Multiplicative scale | 0.717 | 0.433 | 1.189 | 0.197 |
|  |  | RERI | -0.517 | -1.473 | 0.438 | 0.856 |
|  |  | AP | -0.311 | 0.870 | 0.248 | 0.138 |
|  |  | SI | 0.562 | 0.256 | 1.232 | 0.925 |
|  | Yes | Multiplicative scale | 1.064 | 0.554 | 2.044 | 0.852 |
|  |  | RERI | 0.096 | -0.594 | 0.786 | 0.392 |
|  |  | AP | 0.064 | -0.398 | 0.526 | 0.393 |
|  |  | SI | 1.240 | 0.217 | 7.097 | 0.405 |

BMI: body mass index; RERI：Relative Excess Risk due to Interaction; AP：Attributable Proportion due to Interaction; SI：Synergy Index; LCI: lower limit of the 95% confidence interval; UCI: upper limit of the 95% confidence interval
