# Supplementary material for: Joint effects of dietary diversity and intake of edible mushrooms and algae on cognitive impairment among Chinese older adults: a population-based cross-sectional study
Source: Front Nutr. 2026 Oct 22;13:1960150. doi: 10.3389/fnut.2026.1960150 (PMC13761222; doi:10.3389/fnut.2026.1960150)
Supplement: Supplementary file 9 [file Table_7.docx]

**Supplementary Table 7**

**Interaction effect of DDS and EMA intake on cognitive impairment risk in sensitivity analyses**

Supplementary Table 7 Interaction effect of DDS and EMA intake on cognitive impairment risk in sensitivity analyses

| Model | Measures | Estimates | LCI | UCI | P |
| --- | --- | --- | --- | --- | --- |
| Model S1 | Multiplicative scale | 1.023 | 0.780 | 1.342 | 0.871 |
|  | RERI | 0.074 | -0.259 | 0.406 | 0.332 |
|  | AP | 0.049 | -0.172 | 0.270 | 0.332 |
|  | SI | 1.171 | 0.538 | 2.548 | 0.346 |
| Model S2 | Multiplicative scale | 0.830 | 0.559 | 1.232 | 0.355 |
|  | RERI | -0.203 | -0.789 | 0.383 | 0.752 |
|  | AP | -0.130 | -0.502 | 0.242 | 0.246 |
|  | SI | 0.734 | 0.345 | 1.560 | 0.789 |
| Model S3 | Multiplicative scale | 0.922 | 0.700 | 1.215 | 0.564 |
|  | RERI | -0.084 | -0.413 | 0.245 | 0.690 |
|  | AP | -0.068 | -0.333 | 0.197 | 0.308 |
|  | SI | 0.735 | 0.268 | 2.020 | 0.725 |
| Model S4 | Multiplicative scale | 0.770 | 0.505 | 1.173 | 0.224 |
|  | RERI | -0.301 | -0.969 | 0.366 | 0.812 |
|  | AP | -0.182 | -0.579 | 0.215 | 0.184 |
|  | SI | 0.685 | 0.346 | 1.354 | 0.862 |

RERI: Relative Excess Risk due to Interaction; AP：Attributable Proportion due to Interaction; SI：Synergy Index; LCI: lower limit of the 95% confidence interval; UCI: upper limit of the 95% confidence interval
